# Influence of Previous Emergency Department Visit Information on Care of Current Patients

**DOI:** 10.5811/westjem.40047

**Published:** 2025-07-17

**Authors:** Ricardo X. Noriega, Juan Nañez, Emily Hartmann, Scott B. Crawford, Chantel D. Sloan-Aagard

**Affiliations:** * Brigham Young University, Department of Public Health, Provo, Utah; † Paso del Norte Health Information Exchange, El Paso, Texas; ‡ Texas Tech University Health Sciences Center El Paso, Department of Emergency Medicine, El Paso, Texas

## Abstract

**Introduction:**

Past patient data from health information exchanges (HIE) can enhance physician-patient interactions, although how and how often is unclear. We sought to determine how and how often past medical records provided by an HIE impacts current decision-making by emergency physicians.

**Methods:**

We identified qualifying emergency department (ED) visits between September 24–26, 2022. The primary feature of a qualifying visit was a separate ED visit within three days prior at a separate hospital system. Fifty-five charts with essential details of each patient’s most recent visit were reviewed in duplicate by 22 emergency medicine residents. Reviewers accessed prior medical records for each patient via an HIE clinical viewer. The primary outcome was the influence of knowledge from prior records on interactions during the most recent visit, measured with 11 Likert-scale ratings. Reviewer agreement was used as an indicator of confidence.

**Results:**

Reviewers most frequently agreed that the information from the prior visit was valuable “a moderate amount” (25% of all reviewer pairs) and agreed that the information would cause them to change their approach (69%). They would adjust treatment protocols because of understanding what had been tried previously (67%) and ask the patient different questions (78%). There was also agreement that they would further compare laboratory tests or imaging between visits (67%) and better understand patient behavioral patterns (73%).

**Conclusion:**

Access to patients’ previous medical records (diagnoses, imaging reports, discharge reports, etc) via HIEs impacts how emergency physicians communicate with patients, evaluate cases, and make medical decisions.

## INTRODUCTION

Health information exchanges (HIE) have the potential to increase the efficiency and effectiveness of health systems. With the widespread adoption of electronic health records (EHR), the confidential sharing of patient information among clinicians responsible for treating a patient should be simple. However, differences in proprietary EHRs and the way that individual clinicians fill them out, as well as the fact that records may be available only within closed systems mean they are not being used to their full potential.^1^ The HIE harnesses and leverages the benefits of EHRs by connecting multiple organizations and creating a more robust healthcare network, so that clinicians can easily access relevant, timely, and accurate patient information.^2^ Other benefits of HIEs include improved quality, efficiency, cost-effectiveness, emergency response, research, and public health support.^3^–^5^ One setting where HIEs can have a significant impact is the emergency department (ED), where patients often present with urgent and complex conditions that require coordinated and informed care.

Emergency departments have challenges in providing efficient services. These challenges include time limitations, staff shortages, extended stay visits, crowding, low patient satisfaction, and clinical documentation.^6^–^8^ Several observational studies show the benefit of HIEs in emergency settings. Ben-Assuli et al collected 281,750 ED referrals from seven different hospitals. The study concluded that an HIE reduced the number of single-day and seven-day readmissions for all patients. Unnecessary admissions also decreased due to access to past medical history because the clinician did not have to repeat in-depth examinations that had already been recently completed.

A similar study found that between non-affiliated hospital EDs in a region, use of a HIE would lower the cost per patient, as well as reduce overall ED costs, by preventing duplication of lab tests and imaging studies and decreasing ED length of stay and admissions.^9^–^13^ The same studies reported that more than 70% of patients perceived an improvement in the quality of care because of HIE use. Other studies were more specific in studying the impact of HIE in reducing the need for imaging in some of the most common ED chief complaints such as headache and back pain.^14^ Using an HIE was associated with decreased odds of unnecessary diagnostic neuroimaging (odds ratio 0.38, confidence interval [CI] 95% 0.29–0.50) and 64% lower odds of repeated diagnostic imaging for back pain.^15^

Despite evidence of the benefit of using HIEs in EDs, they are not widely adopted, and many emergency physicians with HIE access do not regularly use them because they do not perceive it as important.^16^,^17^ We sought to determine the impact of how accessing an HIE could change emergency physicians’ perceptions and behavior patterns in patient care delivery when they obtain patients’ information from a recent ED visit at another hospital.

## METHODS

We conducted this study in El Paso, Texas, using the Paso del Norte Health Information Exchange (PHIX). The PHIX is available to all clinicians in the area. Medical residents regularly receive login credentials to view patient information via PHIX’s secure portal. The PHIX clinical viewer allows clinicians to access their patients’ health records electronically. The shared data encompasses diagnoses, lab results, radiology and pathology reports, immunizations, medications, and various clinical notes from physicians and nurses, such as discharge summaries, admission history and physicals, progress notes, and consult notes. We used data from PHIX to attempt to recreate the decision-making process when emergency physicians are presented with a new case. Study procedures were approved by the institutional review board at Brigham Young University.

### Recruitment

We enrolled 22 emergency medicine residents from the Texas Tech University Health Sciences Center El Paso. Residents who attended the weekly department meeting were invited to take part in the study. Those who agreed to participate were instructed by the study personnel about the process of conducting the chart reviews. Each patient was reviewed in duplicate. Residents in postgraduate (PGY) years (PGY 1, PGY 2, and PGY 3) participated as reviewers.

**Population Health Research Capsule**

What do we already know about this issue?

*Health information exchanges (HIE) can improve emergency department (ED) care by providing access to prior patient records and reducing redundant tests and admissions*.

What was the research question?

*How does access to prior ED visit data via HIE affect emergency physicians’ decision-making and interactions with patients?*

What was the major finding of the study?

*69% of reviewers agreed that prior visit data would influence their approach during the current visit*.

How does this improve population health?

*Access to prior medical records via HIEs can influence emergency physicians’ communication, case evaluation, and decision-making, potentially improving care efficiency*.

### Inclusion and Exclusion Criteria

We collected the most recent 60 cases from the PHIX system that met the following criteria: 1) visit occurred between September 24–September 26, 2022; 2) the patient visited a hospital-associated ED in the city of El Paso; 3) was discharged with a completed discharge report and 4) visited another hospital-associated ED within three days; and 5) both visits had at least one reported *International Classification of Diseases, 10^th^ Rev*, (ICD-10) code. We did not include patients who went from independent EDs to hospital-associated EDs because those visits are often due to direct referrals for specific treatment. By selecting the most recent cases rather than selecting the cases that met some kind of criteria regarding severity of the case, we aimed to estimate how often the residents would identify the HIE as being helpful for patients in the common situation of visiting multiple EDs within a short period of time.

### Clinical Data Review Procedure

Residents were asked to review two sets of clinical information. The first set included ED charts that were printed out from the records found in the PHIX system. The charts contained the following details: history of present illness; medical history; medications; vital signs; review of systems; physical examination; assessment; diagnosis; and management. We referred to these charts with details of the patient’s most recent visit as “current visit.” The second set of information consisted of details from the prior three-day visit, referred to as the “prior visit,” which was reviewed directly from the online PHIX clinical viewer (see Figure). We used the same terms to describe each visit, including tables and figures. Each patient was assigned an anonymous identifying number for data analysis.

### Survey Instrument

We created a brief survey to be completed by resident reviewers for each reviewed chart using the Qualtrics survey platform (Qualtrics International Inc, Provo, UT). We collaborated with clinical partners and internal members of PHIX with clinical experience to hone the survey questions and ensure clarity. Reviewers entered an assigned personal ID as well as the patient’s anonymous ID. First, reviewers were given up to seven minutes to review the information from the current visit. The seven-minute time window was selected based on tests done with clinical staff who work with PHIX. Seven minutes was estimated to be enough time for a physician to review a patient’s medical history, while not being overlong and delaying patient care.

The reviewers were asked whether this was a case with which they were already familiar. If they answered yes, then they recused themselves from further review. They also were asked to pretend they were managing this patient in real time. Residents responded to questions regarding the current visits and how often they see similar cases, whether a consultation would be required or helpful to the case, and if so, what type of consultation. (The survey is provided as supplemental material.)

The reviewers were then informed that the patient had been in another ED within the prior three days. They were given four minutes to review data from that prior visit via the PHIX clinical viewer. They were then asked four follow-up questions detailing 1) whether there was evidence in the chart from the current visit that the treating physician was aware of the prior visit; 2) five options to describe the relationship between the data from the prior visit and the current visit; 3) 11 Likert scale-based questions describing how the information from the prior visit would influence their interactions, diagnosis, or treatment during the current visit; and 4) “overall, how valuable the information from the prior visit was to treating the patient during their the current visit”, with the options “very little,” “a moderate amount,” and “a great deal.”

### Analysis

We analyzed the data using measures of Kendall W, using the KendallW command in the DescTools package in R v4.3.1 (R Foundation for Statistical Computing, Vienna, Austria). Kendall W is typically calculated as a measure of the reliability of chart reviews when categorizing patients. Herein, we use it as a measure of agreement between reviewers. Variability was expected in how reviewers rated the same chart, as there is subjectivity in the questions, and they were responding from their own perspectives regarding how they would act. However, it was of interest how often the residents did agree with one another as an indicator of the confidence that, yes, further information might cause a physician to respond in a different way.

To analyze the responses to the 11 Likert-scale questions relative to how information from a prior visit might influence physician-patient interactions in the current visit, the Likert-scale answers were collapsed into three groups: 1) strongly or somewhat agree; 2) strongly disagree, somewhat disagree or neither agree nor disagree; or 3) did not answer (NA). These collapsed responses were compared between both reviewers of the same chart. We calculated and reported the percentage of time they agreed. For example, if reviewer 1 strongly agreed with a statement and reviewer 2 somewhat agreed, both would be counted as being in the affirmative on that statement for that patient.

## RESULTS

### Descriptives

A total of 133 surveys were logged. Of these, residents recused themselves six times because they were already familiar with the case. One survey was not fully completed. Four surveys were mistakenly reviewed in triplicate, in which case only the first two completed surveys were kept for analysis, and six were reviewed once (reviewers ran out of time and did not finish). There were 110 complete observations remaining, or 55 patients reviewed in duplicate. Most reviewers completed reviewing charts from 5–6 patients.

When asked how often the reviewers saw similar cases in their practice, 54 (49%) of the 110 reviews were marked as daily, 37 (34%) as weekly, 21 as monthly (19%), one as yearly (0.01%), and two as rarely (less than once a year) (0.02%). When reviewers were asked whether they were likely to request a consultation on a particular case, they said no 101 (92%) times and yes 14 (8%) times. The types of desired consults listed were cardiology, otolaryngology, orthopedics, obstetrics/gynecology, the patient’s surgeon, urology, or vascular. Urology was the only consult mentioned twice.

### Rater Agreement Patterns

The Kendall inter-rater reliability values ranged between 0.44–0.66 (0 means no agreement between reviewers, 1 means perfect agreement), indicating that reviewers agreed with one other a moderate amount. There were mixed levels of agreement between reviewers as to whether there was evidence that the actual treating physician had the information from the prior visit during the current visit. Reviewers agreed that the physician did not have the prior information 16 (29%) times; they agreed that the physician did have the prior information 16 times (29%); and disagreed 21 (38%) times.

Raters agreed that in 20 visits (36%) the most common way the prior and current visits were related was through a presentation of the same symptoms. They agreed that the visits were not related two times (4%). Reviewers never agreed that the visits were related to new symptoms that may have been related to behavioral information described during the prior visit. There was one patient for whom reviewers agreed they had worse symptoms related to a procedure or medication from the prior visit, and none who agreed that there were worse symptoms related to diagnoses from the prior visit. For the rest of the patients (60%), the raters disagreed. The most common disagreement between pairs was for 11 visits (20%) that were related to either the same symptoms or worse symptoms relative to the prior diagnosis.

The Likert-scale questions with the lowest inter-rater reliability (0.44) was: “Please indicate your agreement with each of the below statements regarding [whether] knowing the information from the prior visit would impact your interactions, diagnosis, or treatment during the current visit: “I would adjust treatment or recommendations based on known comorbidities.” The statement with the highest inter-rater reliability (0.66) was under the same category, “I would ask different questions of the patient.”

### Influence on Current Physician-Patient Interactions

As described, the Likert-scale data for the key question relative to how patient-physician interactions would be influenced were collapsed into three main categories of agreement (agreed in the affirmative, disagreed, or agreed in the negative). This helped reduce the possible number of combinations of answers between reviewers. The reviewers gave the same answer most consistently regarding whether they would ask different questions of the patient. The reviewers gave differing answers the most when asked whether they would be more or less likely to keep the patient for observation and when asked whether they would be more or less likely to admit the patient for inpatient care. When reviewers gave the same answer, they were much more likely to give the same answer in the affirmative, either strongly or somewhat agreeing with the question (Table 1).

Reviewers most commonly agreed in the affirmative when reporting they would ask different questions of the patient (78% of pairs) if given the information about the prior visit. The statements on which the reviewers most disagreed were those regarding how likely they were to recommend inpatient treatment or staying in the ED for observation (53% of pairs for each). The same statements regarding inpatient treatment or ED observation also yielded the most frequent agreement in the negative (5% of pairs for each). Agreement in the negative was uncommon across all Likert-scale items. Most reviewer pairs agreed in the affirmative that knowing about the prior visit would change their approach during the current visit (69%), they would further investigate prior diagnosis (65%), further compare lab tests or imaging (67%), understand the behavioral patterns of the patient (73%), change whether they requested additional imaging (56%), and adjust treatment or recommendations based on known comorbidities (55%).

When asked overall how valuable the information from the prior visit was to treating the patient during the current visit, the reviewers agreed it was valuable “a moderate amount” (14 pairs, 25%). They agreed that the prior data was valuable “very little” seven times (13%), and “a great deal” five times (9%) (Table 2). The second most common response pairing was for one reviewer to say, “a moderate amount” and a second reviewer to say, “a great deal” (18% of pairs). Thus, 52% of reviewer pairs found the prior record to be valuable a moderate amount or a great deal.

## DISCUSSION

Our study examined inter-rater agreement and the perceived clinical relevance of prior visit information in ED encounters. A total of 110 complete chart reviews, representing 55 patients, offer perspectives on the variability and consistency in how resident doctors interpret and respond to data from previous emergency visits when making medical decisions during current visits. The findings indicate that while reviewers generally found prior records moderately valuable, the variability in responses reflects the inherent subjectivity in clinical decision-making, particularly in cases where prior patient information might or might not influence treatment decisions. Inter-rater reliability for the question on modifying treatment based on comorbidities was moderate (Kendall W = 0.44), yet the highest reliability was observed for inquiries on altering patient questioning (Kendall W = 0.66). This suggests that while prior visit information is considered valuable by many residents, its influence on clinical decisions varies depending on the type of action being considered.

### Rater Agreement Patterns

Revisits to the ED are a common and costly problem in healthcare systems. They indicate a possible failure in the quality or continuity of care, and they may expose patients to crowding or to experience increased wait times and unnecessary risks of infection or adverse events.^18^,^19^ This group of patients is also described by the term “bouncebacks” and can carry some of the highest risk of misdiagnosis.^20^ In this study, the raters agreed that the most common way the prior and current visits were related in 20 cases (36%) was by presenting with the same symptoms. The ED revisit rate in the US within three days is 8.2%, with 32% occurring at a different institution.^19^ According to a large observational study, revisits can occur for various reasons, such as lack of follow-up care, medication errors, misdiagnosis, or incomplete treatment.^21^ The American College of Emergency Physicians encourages high-quality ED health records to enhance patient care via improved evaluation, management, decision-making, and disposition related to an emergency encounter.^22^,^23^

There were mixed levels of agreement between reviewers as to whether there was evidence that the actual treating physician had the information from the prior visit during the current visit. Emergency clinicians may be able to improve the quality and clarity of notes by clearly documenting when or whether prior records were reviewed. Raters agreed that the physician did not have the prior information 16 (29%) times. The HIE can play a vital role in improving the outcomes and satisfaction of ED patients by increasing access to past ED visit information. Evidence indicates that by enabling access to patients’ medical history, medications, allergies, test results, and other data, HIEs can help emergency physicians make better decisions, avoid duplication of services, coordinate care transitions, and prevent unnecessary hospitalizations.^3^–^5^,^16^ The HIEs can also facilitate communication and collaboration among emergency clinicians and other healthcare professionals involved in patient care.^2^ Other studies concluded that more than 70% of patients perceived improved quality of care when HIE systems are used.^9^,^10^,^12^ The reasons for this improvement could be explained by the handiness of detailed information available through HIEs.

### Influence on Current Physician-Patient Interactions

Physicians use a patient’s medical history to diagnose and treat patients effectively, as well as to prevent potential complications and risks.^24^ A patient’s medical history can reveal the relevant comorbidities and prior disease states for which the patient may or may not be under treatment. A study that explored physicians’ decision-making in hospital readmissions concluded that the main causes of readmission were the lack of communication, inadequate continuity, and poor information flow.^25^ Using an HIE can influence physicians’ decision-making process in various ways, such as improving the quality and safety of care by improving information flow and communication for primary care follow-up.^26^ Additionally, it is believed that HIEs can reduce costs and errors, enhance coordination and collaboration between clinicians, facilitate research and innovation, and reduce avoidable admissions.^27^,^28^

In this study, the reviewers most frequently agreed that the information from the prior visit was valuable a moderate amount, with 52% of reviewer pairs saying the information was valuable a moderate amount or a great deal. They further strongly or somewhat agreed in 69% of cases that the information would cause them to change their approach. They also reported they would adjust their treatment protocols because of understanding what had been tried previously, and they would ask the patient different questions. There was agreement that they would further compare lab tests or imaging between visits and better understand the patient’s behavioral patterns. On the other hand, the reviewers most often gave differing answers when asked whether they would be more or less likely to keep the patient for observation and would be more or less likely to admit the patient for inpatient care. This likely reflects individual practice patterns and comfort with options for treatment and follow-up care and highlights the potential variations in these patterns among clinicians.

A study surveyed 216 emergency physicians and found that 63% believed more than one-quarter of their patients could benefit from a HIE system. And 85% of them also found it difficult to obtain patients’ external information without an HIE, which could take over 60 minutes in some cases. The physicians concluded that having access to currently siloed data at outside institutions could improve patient care and the efficiency of healthcare delivery.^17^ Most of the studies explored in this paper highlighted the benefits of HIEs in preventing duplication of diagnoses or follow-up tests, reducing unnecessary admissions or readmissions, and increasing perceived satisfaction with healthcare services. However, some physicians found HIE systems to be unreliable and disruptive to workflow, which could have been due to structural or design issues.^3^,^29^,^30^ The design of HIEs must be carefully considered to increase effective use.

## LIMITATIONS

The study involved only participants from one teaching hospital, which may limit how broadly the results can be applied. However, the method used in the study could be easily replicated in other settings and HIEs to gather more information. The subset of patients selected for this study is also unlikely to match the general patient population as they were all well enough for discharge from an ED within the prior three days. The indication that 92% of the time the reviewers were not in need of a consultation for the case aligns with the fact that they were stable enough for discharge after their first visit.

In this study, anchoring bias is not necessarily a limitation but rather an intentional component in assessing the value of HIE data in clinical decision-making. The structured study setting prompted residents to consider and potentially alter their approaches based on recent visit data, allowing us to observe the impact of accessible historical information on treatment decisions. We acknowledge that real-world scenarios may present a higher risk of anchoring, particularly in high-stress situations, time-constrained environments, or when patients present with symptoms similar to those in previous encounters. In such cases, residents might default to prior visit information as a cognitive shortcut, potentially overlooking new or contextually unique factors. Thus, while anchoring is less of a concern within this study’s design, understanding its role in actual emergency settings is essential.

The strength of this study is that it generated hypotheses for how HIEs affect physicians’ decision-making and patient interactions when prior patient information is accessible, an area which has been strikingly absent from the current literature. Our study further shows that more research is necessary to identify the factors that influence the adoption and use of HIEs, particularly in emergency situations. These findings can also offer guidance in designing and implementing HIE systems that meet the requirements and expectations of physicians and other stakeholders.

The PHIX, the HIE we used for this study, is structured to provide detailed medical records including physician notes, lab and imaging records, immunizations, and medication histories. Health information exchanges use a variety of data structures and sharing methods. Some offer similar record access to the PHIX while many others do not, choosing instead to highlight or restrict to specific information such as previous ICD-10 codes. It is, therefore, important to note that how and how often HIE access influences patient interactions and treatment will likely vary based on the structure and data provided by the HIE.

## CONCLUSION

The study shows how previous records accessed through a health information exchange significantly impact clinical understanding and influence patient care in the ED. Notably, reviewers were most often in agreement that the information from prior visits allowed them to ask more targeted and relevant questions and adapt their diagnostic approach. Additionally, most reviewers saw value in using prior visit information to compare lab tests, understand patient behavioral patterns, and make more informed decisions regarding imaging and treatment adjustments based on known comorbidities. These positive agreements emphasize the role of HIEs in facilitating more comprehensive and precise patient assessments, lowering the requirement for redundant procedures, and ultimately contributing to more efficient and organized healthcare.

However, the variability seen in inter-rater agreement, particularly in treatment adjustments, suggests a more complex integration of past medical records into therapeutic decisions. This variability underscores that although HIEs offer clear benefits, the development of standardized guidelines and training to staff on how to effectively use the EHR’s features for informed decision-making could further optimize their use. The results of this study contribute to the body of literature on the cost-effective and quality-improving potential of HIEs, reinforcing the need for more widespread adoption and system refinements to maximize their impact. Future research should explore how different HIE structures and training protocols might address these variabilities and further improve clinical outcomes, considering patient-specific needs in diverse healthcare settings.

## Supplementary Information



## Figures and Tables

**Figure:**
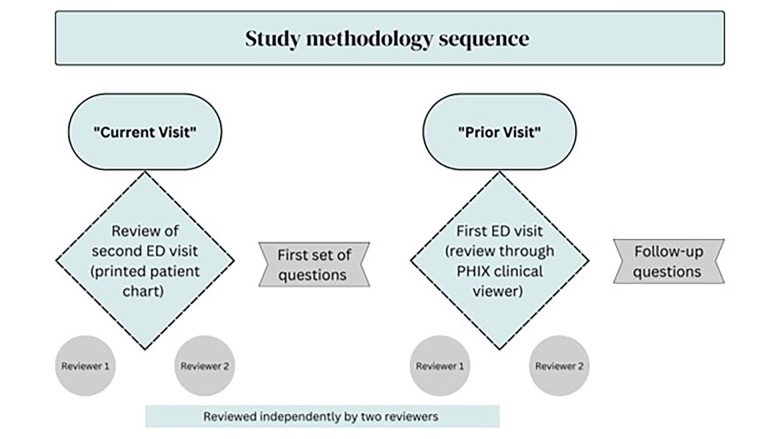
Diagram of the study design. *ED*, emergency department; *PHIX*, Paso del Norte Health Information Exchange in El Paso, TX.

**Table 1:** Frequency and percentage (rounded) of reviewers who gave the same or different answers regarding how the information from prior medical records would impact their interactions and decisions during a current emergency department visit (N=55 reviewer pairs).

Likert Scale Item	Reviewer responses (N, rounded %)		

	Disagreed	Agreed in the affirmative^1^	Agreed in the negative^2^
Knowing what happened in the prior visit would impact my approach during the current visit.	16 (29%)	38 (69%)	1 (2%)
I would ask different questions of the patient.	11 (20%)	43 (78%)	1 (2%)
I would further investigate their prior diagnoses. *(NA=1)*	16 (29%)	36 (65%)	2 (4%)
I would further compare laboratory tests or imaging between visits.	11 (29%)	37 (67%)	2 (4%)
I would adjust my treatment protocol because of understanding what prior medications or treatment were tried.	18 (33%)	37 (67%)	0
I would better understand behavioral patterns of the patient.	15 (27%)	40 (73%)	0
It would change whether I requested additional imaging. *(NA=2)*	22 (40%)	31 (56%)	0
It would change whether I requested additional laboratory tests*. (NA=2)*	27 (49%)	25 (45%)	1 (2%)
I would adjust treatment or recommendations based on known comorbidities.	24 (44%)	30 (55%)	1 (2%)
It would change how likely I was to recommend inpatient treatment.	29 (53%)	23 (42%)	3 (5%)
It would change how likely I was to recommend they stay in the ED for observation.	29 (53%)	23 (42%)	3 (5%)

1 Both reviewers either strongly or somewhat agreed.

2 Both reviewers either strongly or somewhat disagreed, or neither agreed nor disagreed.

*ED*, emergency department; *NA*, missing value.

**Table 2:** Responses recorded by reviewers (N=55 pairs) for the question “Overall, how valuable would the information from the “prior visit” be in treating the patient during the “current visit”? Percents are rounded to the nearest whole number.

	Reviewer 1	Reviewer 2		

		Very little	A moderate amount	A great deal
Overall, how valuable would the information from the “prior visit” be in treating the patient during the “current visit?”	Very little	7 (13%)	5 (9%)	3 (5%)
	A moderate amount	4 (7%)	14 (25%)	5 (9%)
	A great deal	2 (4%)	10 (18%)	5(9%)
