# Supplementary material for: Influence of Previous Emergency Department Visit Information on Care of Current Patients
Source: West J Emerg Med. 2025 Jul 17;26(4):815–22. doi: 10.5811/westjem.40047 (PMC12342408; doi:10.5811/westjem.40047)
Supplement: Supplementary file 1 [file wjem-26-815-s001.docx]

Emergency Room Chart Review

You were given an individual ID number representing yourself. Please enter it here.

________________________________________________________________

On the paper in front of you is a name of a patient and a date when they visited an El Paso Emergency Department (ED). The patient has a study ID number on the paper next to their name. What is the study ID number for this patient?

________________________________________________________________

| Page Break | |  |
| --- | --- | --- |
|  |  |  |

Please read the complete ED chart that has been printed out for this patient. If at any point you think this patient is someone you know (including if you were the physician who treated them), please stop reviewing and proceed to the next question.

You will have up to 7 minutes to review. When the timer hits zero, please continue forward without revisiting the record.

Timer1

| 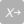 |
| --- |

Do you think this patient is someone you know? (Including if you were the doctor who treated them)

- Yes, I think this is someone I know. (You won't be asked any more information about this patient).
- No, this isn't someone I know.

Skip To: End of Block If Do you think this patient is someone you know? (Including if you were the doctor who treated them) = Yes, I think this is someone I know. (You won't be asked any more information about this patient).

| 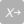 |
| --- |

How often do you see cases in the ED similar to this one?

- Daily
- Weekly
- Monthly
- Yearly
- Rarely (less than once a year)
- Never

| 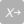 |
| --- |

Imagine that you were the treating physician in the ED and this was your patient. Based on the record, would you likely request a consultation with a specialist?

- Yes, I would consult a specialist to treat this patient.
- No, I would not consult a specialist to treat this patient.

Skip To: instruct_part2 If Imagine that you were the treating physician in the ED and this was your patient. Based on the re... =

|  |
| --- |

What type of specialist would you consult with?

________________________________________________________________

| Page Break |  |
| --- | --- |

The chart you just viewed (what we will call the "Current Visit") was from a patient who also had an ED encounter and/or hospitalization within the **prior three days** (what we will call the "Prior Visit"). 
 
Next, please take a couple of minutes to review a simplified overview of records available from the same patient's "Prior Visit(s)" in the ED and/or hospital within 3 days prior. You will also see a simplified overview of records available from prior encounters more broadly. You will access this data in a simplified format using provided credentials for master.phixnetwork.org. If you need help, please raise your hand and the study personnel will assist you. 


You will be given 4 minutes to review this record. When the counter hits zero, please proceed **without going back to the record**.

Timer2

| Page Break |  |
| --- | --- |

| 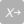 |
| --- |

Is there evidence in the record of the **current visit** (initial chart you reviewed) that the treating physician was aware of **any** important information from the **prior visits**, such as recent procedures or comorbidities?

- Yes
- No

| 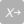 |
| --- |

Which statement below **best** describes the relationship between the data from the "prior visit" (second set of information) and the "current visit" (first chart you reviewed)?

- The prior visit is not related at all to the current visit.
- Patient presented with same symptoms during current and prior visits.
- The patient presented with worsening symptoms related to diagnoses from prior visits.
- The patient presented with worsening symptoms related to procedures and/or medications from prior visits.
- The patient presented with new symptoms that may be related to behavioral information described during the prior visit.

| 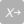 |
| --- |

Please indicate your agreement with each of the below statements regarding if knowing the information from the prior visit would impact your interactions, diagnosis or treatment during the current visit.

|  | Not Applicable | Strongly disagree | Somewhat disagree | Neither agree nor disagree | Somewhat agree | Strongly agree |
| --- | --- | --- | --- | --- | --- | --- |
| Knowing what happened in the prior visit would impact my approach during the current visit. |  |  |  |  |  |  |
| I would ask different questions of the patient. |  |  |  |  |  |  |
| I would further investigate their prior diagnoses. |  |  |  |  |  |  |
| I would further compare laboratory tests or imaging between visits. |  |  |  |  |  |  |
| I would adjust my treatment protocol because of understanding what prior medications or treatment were tried. |  |  |  |  |  |  |
| I would better understand behavioral patterns of the patient. |  |  |  |  |  |  |
| It would change whether I requested additional imaging. |  |  |  |  |  |  |
| It would change whether I requested additional laboratory tests. |  |  |  |  |  |  |
| I would adjust treatment or recommendations based on known comorbidities. |  |  |  |  |  |  |
| It would change how likely I was to recommend inpatient treatment. |  |  |  |  |  |  |
| It would change how likely I was to recommend they stay in the ED for observation. |  |  |  |  |  |  |

| 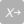 |
| --- |

Overall, how valuable would the information from the "prior visit" be in treating the patient during the "current visit"?

- Very little
- A moderate amount
- A great deal

| 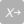 |
| --- |

Is this the last patient you were asked to review today?

- Yes
- No

Skip To: End of Survey If Is this the last patient you were asked to review today? = Yes

Please set aside this patient, and proceed to the next one. You will be asked the same questions about the next patient's ED encounters on the list.

End of Block: Main_questions
